# Psychometric properties of the Persian version of the Cancer attitude inventory

**DOI:** 10.1186/s12889-019-7756-3

**Published:** 2019-10-29

**Authors:** Maryam Khazaee-Pool, Alireza Shoghli, Tahereh Pashaei, Koen Ponnet

**Affiliations:** 1 Department of Public Health, School of Health, Mazandaran University of Medical Sciences, Sari, Iran; 2 Health Sciences Research Center, Addiction Research Institutes, Mazandaran University of Medical Sciences, Sari, Iran; 3 Department of Health Education and Promotion, School of Health, Zanjan University of Medical Sciences, Zanjan, Iran; 4 Social Medicine Department, School of Medicine, Social Determinants of Health Research Center, Zanjan University of Medical Sciences, Zanjan, Iran; 5 Environmental Health Research Center, Research Institute for Health Development, Kurdistan University of Medical Sciences, Sanandaj, Iran; 6 Department of Public Health, Faculty of Health, Kurdistan University of Medical Sciences, Sanandaj, Iran; 7 Department of Communication Sciences, imec-mict-Ghent University, Ghent, Belgium

**Keywords:** Attitude, Cancer, Instrument, Psychometrics

## Abstract

**Background:**

The Cancer Attitude inventory (CAI) was developed to measure attitudes toward cancer. The aim of the present study was to describe the development of the Persian version of the CAI and to evaluate its psychometric properties in an Iranian sample.

**Methods:**

The forward–backward method was used to translate the CAI scale from English into Persian. After linguistic validation and a pilot check, a cross-sectional study was performed and psychometric properties of the Iranian version of the questionnaire were assessed.

The scale validation was conducted with a convenience sample of 820 laypeople. Construct validity was assessed through both exploratory factor analysis (EFA) and confirmatory factor analysis (CFA). Internal consistency was assessed through Cronbach’s alpha analysis and test-retest analysis.

**Results:**

Five factors were identified in CAI: isolation, helplessness, fear of consequence, belief of control and independence, and fear of death. The results achieved from the CFA displayed that the data fit the model: the relative chi-square (× 2/df) = 2.98 (*p* < .001), and the root mean square error of approximation (RMSEA) = .07 (90% CI = .06—.07). All comparative indices of the model had scores greater than .80, demonstrating a good fit to the data. Cronbach’s Alpha and the intra-class correlation coefficient (ICC) were .97, which is well above the acceptable threshold.

**Conclusions:**

The results indicate that the Persian version of the CAI is practical, reliable and valid. Consequently, the instrument could be used in plans to create positive attitudes about cancer control and treatment among Persian people.

## Background

Over the past two decades, cancer incidence rates have increased, particularly in developing countries such as Iran [1]. More than 70% of cancer-related deaths occur in developing countries [2]. At present, cancer is an intricate disease, incurable in many forms. Furthermore, when people think about cancer, their thoughts often include worries about life and death and a fatalistic view about the inevitability of death once diagnosed [3]. As such, people’s attitudes toward cancer have not kept pace with medical progress. Several scholars have stressed the importance of increasing people’s knowledge of and improving attitudes toward cancer prevention, suitable programs, and comprehensive policies and guidelines in developing countries [4].

Public attitudes toward cancer and cancer patients may significantly influence cancer control strategies and improve cancer patients’ quality of life and recovery. Furthermore, the effectiveness of cancer prevention plans is subject to public knowledge of cancer and cancer patients [5, 6]. Overall, studies conducted in developing countries have demonstrated that a large number of people have an insufficient understanding of cancer’s causes, threats, and prevention [7, 8]. People’s beliefs about their ability to perform a particular behavior successfully (i.e., self-efficacy) are important prerequisites for behavioral changes [9], and studying attitudes toward cancer may be supportive in controlling cancer and other chronic diseases (e.g., heart diseases or diabetes).

More specifically, having a positive attitude is a supportive factor for one’s overall health, and optimism affects how one deals with cancer [10]. Researchers have linked people’s fear and ignorance of cancer to their failure to engage in preventive behavior or participate in early detection activities [11]. Treatment that deals with emotions and attitudes can encourage individuals with cancer to think more positively, which in turn affects their quality of life [12]. People with cancer and their relatives may feel embarrassed about their emotional reactions to cancer. Unhappiness, hopelessness, and worry are all normal parts of learning to cope with major life changes. For instance, having a positive attitude toward cancer can influence a person to utilize healthy habits such as performing preventive self-care, increasing physical activity, improving dietary patterns, smoking less, recognizing early warning signs, and seeking early mediation [13]. An early study by Gutteling and colleagues (1987) demonstrated that Dutch women with low levels of fear of cancer knew more about the disease, had greater intentions to behave preventively, had a lower estimation of their chance of getting the disease, and felt that cancer was less threatening than did those with higher anxiety levels [14].

In addition, researchers have linked cultural beliefs to people’s cancer attitudes and screening behaviors [5, 15, 16]. Challenges to cancer control can arise out of cultural beliefs such as myths (e.g., death, fear, pain and suffering, loss of control or independence, helplessness, and isolation) and stigma, and efforts to increase cancer knowledge can be adversely affected by those beliefs. Myths and stigma can affect people’s behaviors in ways such that they are less likely to decrease behaviors that increase their cancer risk or to seek the support and facilities they need when they are diagnosed with cancer [15].

In Iran, obstacles to cancer prevention and screening include a deficiency of community-based events for some cancers—like cervical cancer screening, lack of knowledge, misunderstanding, and unsuitable health behaviors, as well the social and cultural issues stemming from customs, modernism, religious convictions, and bans [16]. A systematic study reported that culture and religion are valuable factors contributing to the scarcity of cervical cancer screening [17]. Many elements formed from people’s socio-cultural attitudes lead them to believe that they are not at risk for cancer [18, 19]. Consequently, knowing people’s beliefs about cancer, prevention, and educational interventions can play a significant role in providing health education and, thus, decreasing mortality due to cancer.

Common folk beliefs about cancer include that surgery spreads cancer and that a bruise or sore causes cancer [20]. Therefore, cultural beliefs are strongly considered significant elements of not only cancer prevention and other health-seeking behavior, but they also influence people’s behavior after diagnosis [21]. Social consequences of being labeled with a cancer diagnosis (e.g., shame) may result in treatment delays [22]. Additionally, attitudes may influence how people cope with cancer. Furthermore, Berrenberg’s study (1991) has been accepted as associating positive attitudes with greater survival rates [23], and meta-analyses support the general notion that optimism and positive attitudes are linked to higher survival rates or better heath in general in people who are cancer-free [24, 25]. On the other hand, some cancer studies refute this hypothesis [26] and point out the potential harm that could be created by encouraging positive attitudes when they are not operative [27].

People who receive cancer diagnoses often feel rejected and isolated [26]. However, thirty years ago, Taylor et al. (1985) linked perceived social support to better adjustment of people with cancer and found that positive attitudes concerning cancer are more likely to occur when people receive social support [28].

Although attitudes are believed to play an important role in cancer management, surprisingly few studies have been devoted to measuring people’s attitudes toward cancer. Furthermore, the use of non-validated questionnaires makes comparison across studies difficult, if not impossible [29]. As such, the lack of a psychometrically sound scale of attitudes toward cancer is an important research gap. One validated instrument that assesses people’s attitudes toward cancer is the cancer attitude inventory (CAI). The CAI, developed by Berrenberg in 1989, consists of 41 items [23]. The original version of the CAI was developed and validated in America [23]; however, the instrument has not yet been tested in Iran. This instrument can assess different aspects of people’s attitudes toward cancer. Furthermore, individuals’ opinions are also influenced by a society’s cultural values, and interest is growing in exploring cultural values and identifying the role that they play in people’s attitudes toward cancer. Knowing people’s attitudes might explain their behaviors, and determining their screening attitudes is important for early diagnosis of cancer [30]. With this in mind, the current study aims to measure the psychometric properties of the Persian version of the CAI in Iranian people.

## Methods

### The Cancer attitude inventory

The CAI is a self-reported scale that consists of 41 items. Higher scores on the CAI indicate a more negative attitude toward cancer. Sample items include “A person is never really cured after cancer” and “Once you have had cancer, you can never be normal again.” Table 2 provides an overview of all items. The items are answered on a 6-point scale ranging from 1 = *strongly agree* to 6 = *strongly disagree* [23].

### Translation procedure

First of all, permission was granted from the developer of CAI to translate the instrument from English into Persian by using a forward-backward translation method. First, two independent expert Persian translators who had no prior knowledge of the CAI translated the instrument into Persian. These translated versions were compared and integrated into a provisional Persian version of the CAI. This version was back-translated into English by two independent individuals who spoke fluent Persian and English and had never seen the English version of the questionnaire. These individuals translated the Persian version back into English, and an interim English version of the CAI was created. Finally, the original English version was compared with the latter version, and a number of additional minor revisions were made to make the Persian version more fluent.

Thereafter, a professional team (four experts in cancer diagnoses and one epidemiologist) compared the original version of the CAI with the back-translated English version. After some cultural and linguistic adaptations, a preliminary Persian version of the CAI questionnaire was prepared. This Persian version was tested in a pilot study with 30 users who had no cancer, and thereafter, the final Persian version of the CAI was solidified.

## Statistical analysis

To test the psychometric properties of the Persian version of the CAI, we examined the face validity, content validity, construct validity, and reliability of the instrument.

### Face validity

The face validity of a scale determines the extent to which the scale seems valid. Thus, the establishment of face validity should be prioritized as the first step in the scale validation process [26]. In this study, both qualitative and quantitative face validity were implemented. A group of laypeople (*n* = 10) was asked to evaluate each item of the CAI and indicate if they felt ambiguity or difficulty in replying to the items of the Iranian version of the CAI. In the quantitative phase, the item impact score (importance × frequency) was assessed to calculate the percentage of laypeople who considered each item of the CAI important or quite important on a 5-point Likert scale. Items with an impact score below 1.5 were considered inappropriate (which indicates correspondence with a mean frequency of 50% and a mean importance of 3 on a 5-point Likert scale). Items that met such quantitative criteria were kept in the instrument; other items were deleted [31]. Generally, all items had impact scores ranging from 1.6 to 5. As such, the first version of the CAI consisted of 41 items.

### Content validity

Both qualitative and quantitative content validity of the CAI were examined after the face validity. For qualitative content validity, a panel of 10 scientific experts, including five health education experts, four cancer experts, and one epidemiologist, assessed the wording, grammar, and scaling of the CAI. For quantitative content validity, both the content validity index (CVI) and content validity ratio (CVR) were calculated. The experts were asked to indicate the relevance, simplicity, clarity, and ambiguity of each item (i.e., the CVI) [13, 32]. To calculate the CVI, a 4-point Likert scale was applied. The answers were rated from 1 = *not relevant, not clear, and not simple* to 4 = *very relevant, very clear, and very simple*. The CVI was calculated as the number of items that received a score of 3 or 4 from the experts [13, 33]. In other words, the CVI of each item is the proportion of experts who rated the item content valid (with a score of 3 or 4) [13, 31–33]. A CVI score of .80 or above for an item was considered acceptable [31]. The items’ necessity was evaluated by the CVR, as the expert panel rated each item from 1 to 3, where 1 = *essential*, 2 = *useful but not essential*, and 3 = *not essential* [13]. The CVR of each item was assessed using the following formula: CVR = [Ne – (N/2)] / (N/2). In this formula, Ne indicates the number of experts who score the calculated item as “essential,” and N is the total number of panelists in the expert panel. To decide the cutoff point and numeric value of CVR, Lawshe’s table was applied [34]. Based on the Lawshe table, items with CVR scores lower than .62 were not considered acceptable and were deleted.

### Construct validity

The construct validity was tested using exploratory factor analysis (EFA), confirmatory factor analysis (CFA), and item-scale correlation for the 41 CAI items after testing their face and content validity.

#### Design and data collection

To test the psychometric properties of the CAI in an extensive setting, a cross-sectional study was conducted in Zanjan, Iran, in November 2017. The study population was healthy laypeople aged 20–50 years. The research environment was public places, including cultural centers, parks, universities, and so on, that provide easy access to research samples. With this aim, a convenience sampling method was used. People who were in public places were approached. If they could read and write in Persian, they were asked to participate. The sample size was estimated a priori. As concluded by Gable and Wolf (2012), a sample of five to ten persons per item is required to ensure a theoretically clear factor structure for EFA and CFA [35]. The desired minimum sample size was thus determined to be 410 laypeople for each phase of construct validity (41 items × 10 participants). In all, 820 laypeople participated for EFA (410 laypeople) and CFA (410 laypeople). These participants were approached in public places and invited to test the construct validity of the CAI. After the interviewers provided information about the aim of the study, people who decided to join completed the CAI. The participants were guaranteed that their answers would remain anonymous and confidential and that they could retract their contribution at any given time. After that, participants’ written consent was obtained. In addition, people’s demographic characteristics, including age, education level, gender, and marital status were asked (see Table 1). The CAI took 30 to 40 min to complete.

**Table 1:** Characteristics of the study sample

		EFA sample (*n* = 410)	CFA sample (*n* = 410)	Test-retest sample (*n* = 40)
		Number (%)	Number (%)	Number (%)
Age (years)				
	20–29	114 (27.8)	104 (25.37)	8 (20)
	30–39	221 (53.9)	211 (51.46)	23 (57.5)
	40–54	75 (18.3)	95 (23.17)	9 (22.5)
Gender				
	Male	55 (13.41)	45 (10.98)	17 (42.5)
	Female	355 (86.59)	365 (89.12)	23 (57.5)
Educational Level				
	Primary	84 (20.49)	130 (31.71)	7 (17.5)
	Secondary	232 (56.58)	188 (45.8)	25 (62.5)
	Higher	94 (22.92)	92 (22.43)	8 (20)
Marital status				
	Single/divorced/ widowed	293 (71.47)	327 (79.24)	24 (60)
	Married	117 (28.53)	83 (20.24)	16 (40)

#### Exploratory factor analysis

After content validity, the construct validity of 41 items was examined by performing EFA to detect the main factors of the CAI. For this purpose, 410 lay people were selected in public places of Zanjan using a convenience sampling method.

In this case, a principal component analysis (PCA) with varimax rotation was applied to extract the main factors of CAI. Furthermore, to assess the sampling adequacy for the factor analysis, the Kaiser-Meyer-Olkin (KMO) measure and Bartlett’s test of sphericity were applied [36]. Any factor with an eigenvalue equal to 1 or above was considered significant for factor extraction. Furthermore, a scree plot was used to specify the number of factors. An acceptable score for factor loadings was considered equal to or greater than .40 [37].

#### Confirmative factor analysis (CFA)

In order to assess the coherence between the data, a CFA was applied. Therefore, 410 lay people were selected using a convenience sampling method. The model fit was evaluated by using multiple fit indices. The model fit was evaluated using several fit indices, including relative chi-square, goodness-of-fit index (GFI), normed fit index (NFI), comparative fit index (CFI), root mean square error of approximation (RMSEA), and standardized root mean square residual (SRMR) [38]. Relative chi-square is the chi-squared ratio to degrees of freedom, and a value below 3 is considered to be satisfactory [39]. The GFI, CFI, and NF range from 0 to 1; however, values ≥ .90 are commonly indicated as acceptable model fits [40]. An RMSEA value between .08 and .10 shows an average fit, and a value below .08 demonstrates a good fit. Still, in recent studies there is agreement by experts on our topic that a value between .06 and .07 is more appropriate [41]. Values below .05 indicate a good fit for SRMR, but values between .05 and .08, and between .08 and .10 indicate a close fit or are acceptable, respectively [42].

#### Item-scale correlation

Lastly, item-scale correlation was applied to measure the degree to which each CAI item was correlated to its hypothesized subscale. Pearson correlation coefficient values equal to or above 0.4 were considered satisfactory [43].

### Reliability

#### Internal consistency

Cronbach’s’ alpha coefficient was applied to assess internal consistency of the whole scale and individual dimensions of the CAI questionnaire. The acceptable level of alpha values was 0.70 or higher [43, 44].

#### Test-retest

Test-retest reliability was applied to examine the questionnaire’s stability by estimating the intra-class correlation coefficient (ICC). A separate sample of laypeople (*n* = 40) completed the CAI twice with a 2-week interval. ICC values of .40 or above were considered acceptable [44]. SPSS version 23.0 was used in order to perform the statistical analyses [45].

## Results

### Construct validity

#### Exploratory factor analysis (EFA)

**Fig. 1:**
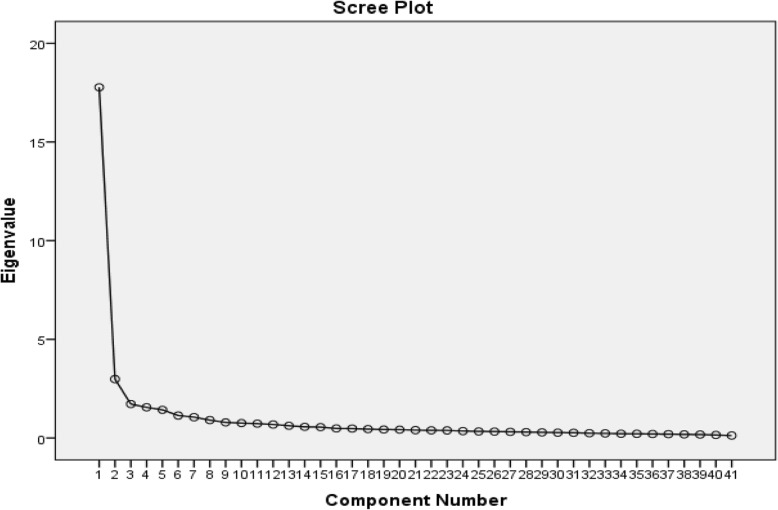
Scree plot for determining factors of the CAI

The KMO measure was .953, and Bartlett’s test of sphericity was significant (χ2 = 11,008.177, *p* < .001), indicating adequacy of the sample for EFA. A five-factor solution for the 41-item instrument was revealed based on eigenvalues greater than 1 and factor loadings equal to 0.4 or above. The five-factor solution jointly explained 62.077% of the total observed variance (Table 2). The scree plot also showed a five-factor solution (see Fig. 1). The five factors were named according to the items that loaded the highest on each construct.

**Table 2:** Exploratory factory analysis of the CAI (*n* = 410)

Item	Factor 1	Factor2	Factor3	Factor4	Factor5
14. People with cancer just waste away	**.725**	.243	.067	.195	.272
23. Cancer is almost always terminal	**.716**	.174	.212	.196	.195
25. Having cancer usually ruins one’s career	**.708**	.220	.183	.180	.106
26. Cancer is a topic most people want to avoid.	**.708**	.100	.228	.166	.114
18. If someone I loved had cancer, it would be difficult for me to talk to him/her about their experiences with the disease	**.703**	.186	.095	.230	.277
38. Cancer usually ruins close personal relationship	**.703**	.147	.195	.201	.169
33. Having cancer permanently changes your whole life	**.702**	.215	.257	.102	.077
36. A person with cancer is helpless	**.698**	.121	.292	.152	.097
13. Cancer can ruin a family	**.679**	.287	.261	.125	.126
39. Cancer devasted the lives of those it touches	**.672**	.168	.010	.104	.209
12. People with cancer don’t want to talk much about their experiences with the disease	**.523**	.342	.358	.367	−.110
2. People with cancer don’t want others to know they have the disease	**.483**	.369	.300	.411	−.155
6. Getting cancer would be one of the two or three worst things that could happen to me in my life	.226	**.772**	−.005	.146	.096
15. A person is never really “cured” of cancer	.227	**.762**	.114	.114	.235
16. Compared with other diseases, there is something uniquely sinister (evil) about cancer	.150	**.752**	.175	.098	.236
5. Cancer usually means financial ruin for patients and their families	.167	**.735**	.157	.129	.041
28. Long term survival (more than 7 years) of cancer is a rare event	.153	**.710**	.207	.167	.187
10. Cancer is an unpredictable disease	.164	**.689**	.206	.043	.197
30. Cancer almost always results in some kind of disfigurement	.212	**.688**	.272	.239	.106
29. Cancer almost always means severe pain	.184	**.685**	.257	.232	.121
11. If I had to choose, I would rather have diabetes than cancer	.172	**.633**	.236	.014	.162
41. Cancer is an ugly disease	.136	**.547**	.361	.310	.046
31. If I had to choose, I would rather have heart disease than cancer	.092	**.541**	.255	.259	−.009
1. I worry a lot about getting cancer	.260	.298	**.706**	.134	.173
22. It makes me uncomfortable to think about cancer	.248	.160	**.670**	.165	.129
4. Cancer is more frightening than most other diseases	.196	.354	**.639**	.133	.119
34. The word “cancer” makes my skin crawl	.286	.350	**.634**	.159	.164
17. Of all the diseases I would be likely to get, I am most afraid of cancer	.276	.261	**.585**	.205	.224
24. Cancer is more frightening than heart disease	.220	.197	**.511**	.119	.359
21. Having cancer can give new meaning to life	.092	.134	.169	**.662**	.360
20. One day soon there will be a cure for cancer	.137	.085	.127	**.656**	.367
27. People who have been cured of cancer are no different from people who have never had the disease	.291	.186	.206	**.627**	.141
37. A person who has had cancer can live a fully satisfying life	.442	.295	.165	**.614**	.014
3. Positive things can be gain from having cancer	.229	.237	.278	**.580**	.259
40. The world can learn a lot from people who have or have had cancer	.342	.197	.031	**.556**	−.092
35. Having cancer provides a person with a unique opportunity for personal growth	.389	.184	.051	**.497**	.164
19. Getting cancer means having to mentally prepare oneself for death	.258	.260	.195	.238	**.687**
32. It is depressing to be around someone with cancer	.270	.316	.195	.221	**.632**
9. Once you’ve had cancer, you can never be “normal” again	.321	.341	.317	.182	**.568**
8. For the most part a diagnosis of cancer is the death sentences	.287	.370	.390	.240	**.494**
7. People with cancer usually develop psychological problems	.279	.308	.426	.132	**.465**

Note: Figures in bold are related to factor loadings equal to or greater than 0.40

*Items 3, 20, 21, 27, 35, 37, and 40 are reversed scored

As presented in Table 2, five factors were found. Factor 1 (isolation) included 12 items (items 2, 12–14, 18, 23, 25, 26, 33, 36, 38, and 39); Factor 2 (helplessness) included 11 items (items 5, 6, 10, 11, 15, 16, 28–31, and 41); Factor 3 (Fear of consequence) included 6 items (items 1, 4, 17, 22, 24, and 34); Factor 4 (belief of control and independence) included 8 items (items 3, 4, 20, 21, 27, 35, 37, and 40); and Factor 5 (fear of death) included 5 items (items 7–9, 19, and 32). Refer to Appendix 1 for CAI items.

#### Confirmatory factor analysis (CFA)

**Fig. 2:**
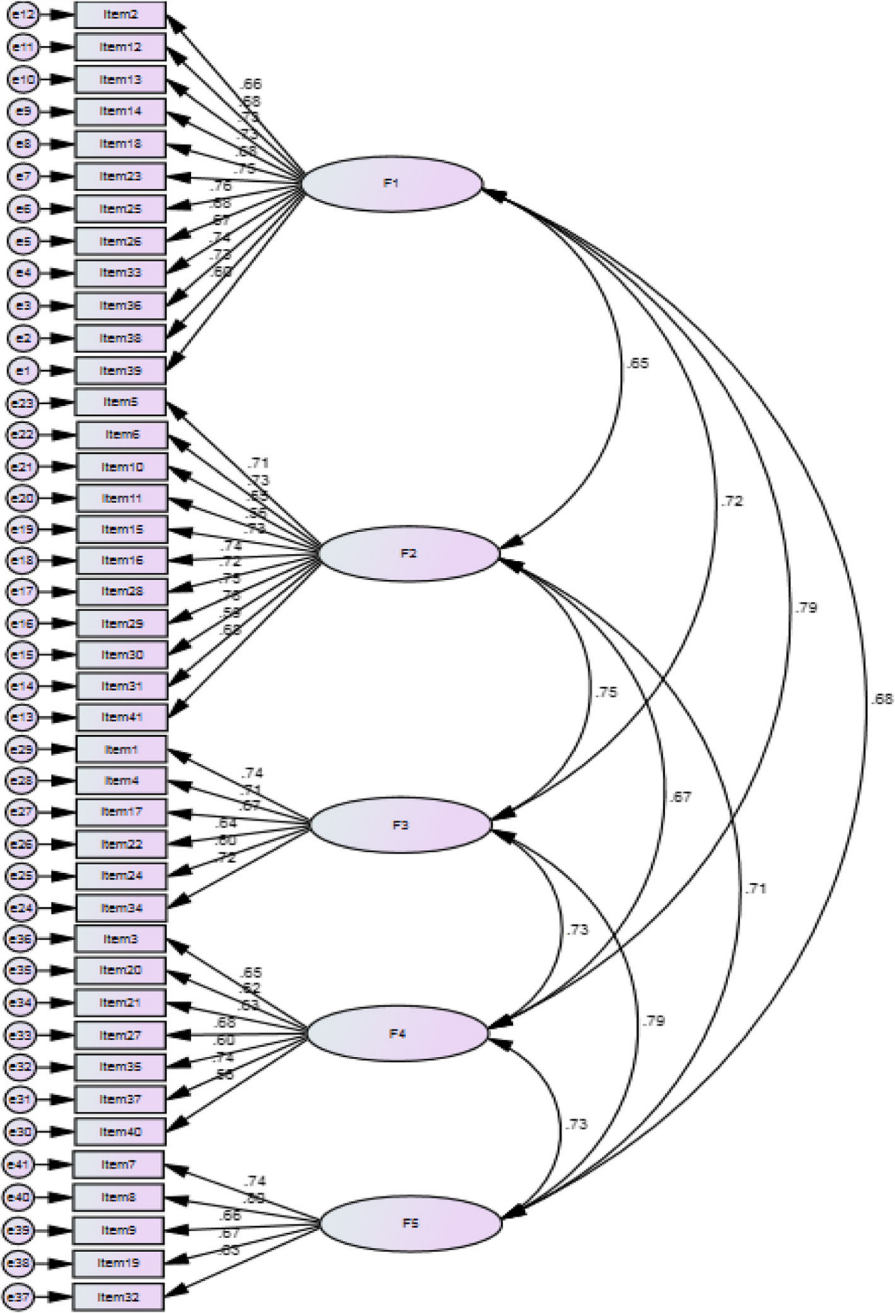
A five-factor model for the scale gained from confirmatory factory analysis (*n* = 410)

A CFA was conducted on the 41-item scale in order to assess the fitness of the model obtained from the EFA. Covariance matrixes were used and fit indices were calculated. Figure 2 demonstrates the fit of the model. All fit indices proved to be good. The relative chi-square (χ2/df) was equal to 2.98 (*p* < .001). The RMSEA of the model was .07 (90% CI = .06–.07), and the SRMR was .050. All comparative indices of the model, including GFI, AGFI, CFI, RMR, NFI, and NNFI, were more than .80 (.85, .82, .83, .89, .87, and .89 respectively), demonstrating a good fit to the data.

#### Item-scale correlation

The item-scale correlation for the CAI is shown in Table 3. All coefficients are higher than .40, and most of them are higher than .50. Isolation and Fear of Death had the lowest and the highest item-scale correlations, respectively. All items were correlated with their own hypothesized subscales, giving additional support for the construct validity of the scale (Table 3).

**Table 3:** Item-scale correlation matrix for the Five CAI measures (n = 410)

CAI Dimensions I (item number)	I	H	FC	BCI	FD
**I (item number)**					
Item 14	.789	.488	.489	.545	.567
Item 23	.794	.466	.549	.560	.534
Item 25	.784	.467	.520	.540	.488
Item 26	.781	.460	.499	.573	.547
Item 18	.772	.483	.495	.509	.472
Item 38	.791	.443	.518	.491	.476
Item 33	.791	.498	.519	.496	.498
Item 36	.786	.412	.504	.530	.489
Item 13	.664	.464	.469	.403	.434
Item 39	.789	.528	.570	.510	.544
Item 12	.750	.543	.599	.598	.502
Item 2	.714	.540	.566	.603	.472
**H (item number)**					
Item 6	.454	.768	.434	.434	.449
Item 15	.499	.820	.520	.452	.567
Item 16	.444	.809	.538	.441	.563
Item 5	.484	.559	.772	.434	.510
Item 28	.459	.783	.544	.473	.535
Item 10	.428	.719	.526	.407	.507
Item 30	.520	.805	.583	.510	.566
Item 29	.502	.793	.561	.499	.542
Item 11	.420	.718	.485	.446	.483
Item 41	.465	.734	.536	.474	.518
Item 31	.471	.689	.407	.483	.412
**FC (item number)**					
Item 1	.539	.565	.806	.471	.591
Item 22	.502	.430	.775	.448	.492
Item 4	.496	.569	.771	.449	.516
Item 34	.572	.600	.785	.503	.607
Item 17	.555	.531	.792	.515	.563
Item 24	.477	.449	.731	.449	.574
**BCI (item number)**					
Item 21	.411	.419	.434	.745	.474
Item 20	.416	.473	.406	.735	.447
Item 27	.544	.441	.492	.767	.486
Item 37	.653	.514	.522	.790	.529
Item 3	.522	.505	.540	.741	.533
Item 40	.453	.451	.418	.634	.428
Item 35	.528	.468	.484	.672	.453
**FD (item number)**					
Item 19	.502	.514	.533	.522	.830
Item 32	.520	.542	.537	.505	.824
Item 9	.575	.573	.622	.555	.852
Item 8	.587	.622	.651	.584	.848
Item 7	.535	.546	.639	.492	.795

Note^1^: I: Isolation, H: Helplessness, FC: Fear of consequence, BCI: Belief of control and independence, FD: Fear of death

Note^2^: The bold data reflect the higher item-scale correlation for the five structures of CAI questionnaire

### Reliability

Cronbach’s alpha was calculated separately for CAI as well as for each factor of the CAI. Cronbach’s alpha coefficient for CAI was .97, which showed excellent and high internal reliability. Thus, no items of the instrument were removed in this step. In addition, test-retest analysis was performed to assess the stability of the CAI. The results showed satisfactory results. Intra-class correlation (ICC) was .97 for the CAI, providing support for the stability of the scale (Table 4).

**Table 4:** Measures of internal consistency and stability

Factor	The name of factor	Number of items	Cronbach alpha (*n* = 410)	ICC (*n* = 40)
1	Isolation	12 items (2, 12–14, 18, 23, 25, 26, 33, 36, 38, and 39)	.93	.93
2	Helplessness	11 items (5, 6, 10, 11, 15, 16, 28–31, and 41)	.92	.95
3	Fear of consequence	6 items (1, 4, 17, 22, 24, and 34)	.86	.92
4	Belief of control and independence	8 items (3, 4, 20, 21, 27, 35, 37, and 40)	.85	.82
5	Fear of death	5 items (7–9, 19, and 32)	.88	.83
Total		41 items	.96	.97

## Discussion

In the present study, we described the translation and psychometric properties of the Iranian CAI, an instrument to measure attitudes toward cancer that might have an impact on the control and treatment behaviors for cancer. Overall, the results proved that the translated CAI is an appropriate and valid scale that can be used to assess attitudes toward cancer among individuals who speak Persian.

Based on the results, the CVI and the CVR indicated good content validity. Furthermore, consistent with the original CAI [23], Cronbach’s alpha and the intraclass correlation for the Persian version of the CAI were suitable and indicated good reliability and stability. This study presented a five-factor solution for the Persian version of the CAI, including isolation, helplessness, fear of consequence, belief of control and independence, and fear of death. The five factors were able to predict 62.077% of the total observed variance. The explained variance is higher than that found in the original CAI study, in which a one-dimensional factor was found that accounted for 43% of the observed variance [23]. Our explained variance is also higher than that found by Berenberg (1991), who found a two-factor structure: Factor 1 explained 36% of the total variance for 36 items, and the remaining five items loaded slightly on Factor 2, which accounted for only 7% of the variance.

The Farsi version of the CAI consisted of 41 items and resulted in five conceptual dimensions. The resulting five-factor solution (isolation, helplessness, fear of consequence, belief of control and independence, and fear of death) produced in the present study is sufficient for understanding attitudes toward cancer, although the solution is different from the original version of the CAI [23], which showed a unidimensional possible explanation for different aspects of beliefs and attitudes being relevant to cancer screening behaviors [5, 6, 12, 15, 46]. The difference between our results and those of the original version might be related to the nature of the sample, values, culture, and other socioeconomic elements. Future studies in different countries would further lend credibility to the present findings. In intervention and prevention campaigns, highlighting these five factors might be useful so that people become more aware of their attitudes toward cancer, which in turn might have a positive impact on how they perceive treatment.

The relationships between attitudes toward cancer and fear of death, fear of consequence, helplessness, and isolation grow out of common sense. Still, providing factual knowledge about cancer is not sufficient to change people’s attitudes toward cancer. Fear of cancer and its consequences, such as severe illness or death, result in psychological anxiety and tension, which in turn affect people’s quality of life, decisions about whether to continue cancer treatment, and how they live the remaining days of their lives [47].

Individuals respond in various ways when they have to deal with anxiety about death or consequences of disease. Some people may increase health-control behaviors, while others decrease these behaviors [48, 49]. Despite medical progress in cancer control, cancer patients still often experience a lot of pain. Increasing people’s awareness about and improving their attitudes toward life after cancer may impact their approach to handling the concerns and treatment [50].

We also conducted a CFA to determine whether there was coherence between the hypothetical construction of the CAI and data. The CFA delivered good fit indices for this model, and the convergent validity of the five factors of the CAI was suitable. These findings are consistent with the results of the original study [23], indicating that the CAI is reliable when used on Persian-speaking individuals. Additionally, the internal consistency of the CAI revealed suitable reliability for all five factors of the CAI, which is consistent with the original study [23]. Furthermore, after examining 40 persons over a 2-week period, our results revealed that the CAI has good stability in the short term. However, it remains to be demonstrated whether the CAI is still stable in the long term.

### Limitations

This study has some limitations. First, regarding the participants, we only engaged Iranian people without cancer, which possibly limited the external validity of this scale. Although healthy people vary both across and within countries as much as the general public [5, 46], we assume that our convenience sample may be representative of the general population given that people have different beliefs, attitudes, and values toward any type of cancer. Still, for future studies to examine the reliability and validity of the instrument in a sample of adults with different backgrounds and from various regions might be interesting. In addition, future researchers might be interested in incorporating additional scales that measure people’s attitudes toward cancer so that the criterion validity of the Persian version of the CAI can be assessed.

Other limitations related to the nature of survey designs is that respondents may not feel encouraged to provide accurate, honest answers or that they might not feel comfortable providing answers that present themselves in an unfavorable manner. Another limitation of the present research is linked to its sample size and its generalizability. The present sample was limited to a convenience sample of 820 laypeople, and whether we would achieve the same outcomes if we recruited a large representative group of both healthy people and cancer patients is unknown. As such, the present study is unable to measure differences in healthy people and cancer patients. Future studies should aim to include both groups to measure whether attitudes toward cancer and controlling behaviors are similar among healthy people and cancer patients and whether being healthy or having cancer influences individuals’ responses to treatment.

## Conclusion

Our study demonstrated that the psychometric properties of the Persian version of the CAI are appropriate. We believe the Persian version of the CAI may be helpful for healthcare groups to plan health methods that are practical and targeted to specific situations. Further studies in healthy persons and cancer patients are suggested to establish the psychometric properties of the Iranian version of the CAI.

## Data Availability

The datasets produced and analyzed throughout the present study are not publicly available in order to keep the participants’ privacy but are available from the corresponding author on sensible request.

## Abbreviations

CAI: Cancer Attitude Inventory
CFA: Confirmatory factor analysis
CFI: Comparative Fit Index
CVI: Content validity index
CVR: Content validity ratio
EFA: Exploratory factor analysis
GFI: Goodness of Fit Index
ICC: Intraclass correlation coefficient
NFI: Normed Fit Index
NNFI: Non-Normed Fit Index
RMSEA: Root Mean Square Error of Approximation
SRMR: Standardized Root Mean Square Residual
